# Delay in Reaching Institutional Delivery Service Utilization among Mothers Attending Jimma Medical Center, Ethiopia

**DOI:** 10.4314/ejhs.v32i4.2

**Published:** 2022-07

**Authors:** Samira Awel, Vinod V Bagilkar, Bekana Fekecha

**Affiliations:** 1 School of Nursing, Institute of Health, Faculty of Health Sciences, Jimma University, Ethiopia; 2 School of Midwifery, Institute of Health, Faculty of Health Sciences, Jimma University, Ethiopia

**Keywords:** Delay in reaching, Institutional delivery, Jimma-Ethiopia

## Abstract

**Background:**

Maternal delay in the utilization of delivery services is one of the contributing factors for high maternal mortality in developing countries. However, it is preventable by timely arrival for obstetric care. The difference between life and death in obstetrics might be a matter of timely arrival and management. The objective of this study was to assess factors associated with maternal delay in reaching institutional delivery service utilization among mothers attending Jimma Medical Center.

**Method:**

Facility-based cross-sectional study design was employed. The sample size was determined by a single population proportion formula and entered into epi data version 3.1, then exported to SPSS version 23. The data was presented using texts and tables. Binary and multivariable logistic regression analysis with 95% CI for odds ratio (OR) was used to assess the factors.

**Result:**

The prevalence of maternal delay in reaching institutional delivery service utilization was 163(40.2%). Low husbands' educational levels were significantly associated with delay in reaching: illiterate (AOR=4.22, 95% CI: 1.10–16.19), primary (AOR=3.88, 95% CI: 1.24–12.1). Mothers who live in rural areas have been delayed 2 times more than mothers who live in urban areas (AOR=2.22, 95% CI: 1.044–4.73). Likewise, mothers who live a long distance from health facilities are 13 times more likely to delay than mothers who live ≤ 10 kilometers (AOR= 12.89,95%CI:6.66–24.94).

**Conclusion:**

Delay in reaching institutional delivery service was high. Factors such as husband's education, distance of health facility, and living area were significant factors with delay in reaching.

## Introduction

Maternal mortality from complications relating to pregnancy and childbirth in women's lifetime is higher in developing countries (1). Two-thirds of maternal deaths occurred in sub-Saharan Africa. Even if the majority of maternal mortalities can be prevented, by early recognition and treatment, different factors can hinder women's access to emergency obstetric services. According to the EDHS report, maternal deaths account for 25 % of all deaths in women aged 15 – 49, and the maternal mortality ratio was estimated to be 412 maternal deaths per 100,000 live births (2, 18).

Pregnant mothers need to get frequent and timely care, especially during labor and delivery. Because delivery is the shortest and most severe period. It is also known that the time between the beginning of an obstetric emergency and ultimate care is critical for the survival of the mother and her baby, never less, developing countries had a lower chance for accessing emergency obstetric care due to social, cultural, socio-economic, distance, transportation, political constraints, ignorance and female decision making power which may result in maternal delay (3, 4).

Several factors were found to delay access to and utilization of emergency obstetric care among women in sub-Saharan Africa. These barriers are interdependent and occur at multiple levels either at home, on the way to health facilities, or at the facilities (5, 17). The second delay or delay in reaching refers to after a decision is made at home to seek medical care in one of the accessible health facilities, several other barriers may impede access to health facilities (6, 7).

In Ethiopia, where the religious impact, cultural fences, gender inequality in household decision making, and bad experiences, unattainable health facilities are quite common and as the majority of women live in rural areas are likely illiterate, had no approval from their husbands and families and had poor knowledge of the danger signs of pregnancy. Thus, pregnant women usually come to health facilities with obstetric complications due to their delay in appropriate care (8).

On the other hand, Ethiopia has been making substantial improvements on reducing maternal mortality and has attained its Millennium Development goals (MDG) (to reduce maternal mortality) goal of 350 maternal deaths per 100,000 and now stands at 353 in 100,000 according to the 2013 United Nation (UN) and Ethiopian ministry of health estimation 2019 (9).

Despite current increased efforts in Ethiopia, including promoting delivery by health care providers, much more needs to be accomplished for the reduction of maternal mortality. If mothers access health facilities easily, mothers can be saved from preventable deaths as the majority of maternal deaths are preventable. The maternal delay in reaching institutional delivery service utilization is not studied well: especially in the specific study area. In response to this, the study aimed to assess maternal delay in reaching for delivery, service utilization, and associated factors among mothers attending delivery at Jimma Medical center (JMC).

## Methods

**Study design and setting**: The study was conducted at Jimma medical center (JMC) from April 1 to April 30, 2019. JMC is one of the oldest public hospitals in the country. It is located in Jimma town, 352 km southwest of Addis Ababa, capital of Ethiopia. A facility-based cross-sectional study design was employed.

**Study population**: All mothers delivered at Jimma Medical Center (JMC) were a source of population. Those mothers available to utilize the delivery service at Jimma Medical Center (JMC) during the data collection period were considered.

**Inclusion and Exclusion criteria**: Mothers who were present at Jimma Medical Center (JMC) for delivery service during the data collection period and giving consent to participate were included. Mothers who were in severe medical condition, unable to respond, women who utilized a maternal waiting home, and those who were admitted to the ward prior to labor and stayed until delivery were excluded.

**Sample size determination**: The sample size was determined by using the single population proportion formula. The prevalence of maternal delay for utilizing institutional delivery was 40.1% (P=0.401) that was taken from a facility-based cross-sectional study conducted in Hadiya, southern Ethiopia (10). Standard normal distribution of the Z-value at 95% confidence interval (CI) =1.96, and 5% margin of error became 369, and by adding none respondents rate 10% of sample size, the final sample size was 406. All mothers who presented to receive the services of delivery at Jimma Medical Center (JMC) during the data collection period were included consecutively until the required sample size was obtained. This study uses the method of Awel S. et al. and the methods description partly reproduces their wording (20).

Delay in reaching was dependent variable and was tdefined as he time interval from starting to reach health facilities after a decision has been made. Time taken ≥1 hour to reach a facility is considered as delay and less than an hour is considered as no delay.

The following were independent variables.

**Socio-economic factors**: Maternal age, educational status of the mother, educational status of the husband, decision-making power, place of residence, monthly income, mothers, and husband's occupation

**Obstetric related factors**: Parity, ANC follow-up, ANC frequency, history of abortion, maternal knowledge on danger signs, and birth preparedness.

**Accessibility of the health facility**: Distance, cost of transportation, transportation availability, accessibility of road

**Data collection instrument and procedures**: A structured questionnaire was adapted from a similar study conducted in southern Ethiopia and the survey tools developed by JHPIEGO Maternal and Neonatal Health Program (14, 19). The adapted English questionnaire was translated into Afaan, Oromo, and Amharic, and then it was retranslated back into English by two independent language experts to check its consistency. Data were collected through a face-to-face interview by four diploma midwives and one BSC nurse was recruited for supervision.

**Data quality assurance**: To avoid confusion and to ensure a common understanding of the study, data collectors and supervisors were trained regarding the objectives of the study, data collection methods, and the significance of the study. To check the clarity of the tool, pretests were done among 5% of the total sample at another public institution with similar characteristics to the study population and modifications were done on unclear questions. Throughout the data collection process, interviewers were supervised, and regular meetings were held between the data collectors, the supervisor, and the principal investigator to discuss and address any issues that arose during the interviews. Before entering data, the collected data were reviewed and checked for completeness.

**Data analysis and presentation**: The collected data were entered, cleaned, coded into Epi Data version 3.1 statistical software, and then exported to SPSS Windows version 23 for further analyses. Descriptive statistics, binary, and multivariable logistic regression analyses were performed. All variables were entered into binary logistic regression and those explanatory variables with a p-value ≤ 0.25 were considered as a candidate for multivariable logistic regression and odds ratios with 95% CI were computed and variables having a p-value less than 0.05 in the multivariable logistic regression model were considered as significantly associated with the dependent variable. Multi-collinearity between the candidate variables was checked and model fitness was checked using Hosmer and Lemeshow goodness of fitness test. Finally, the results of the analyses were presented in text, tables, and graphs accordingly.

**Ethical Approval**: Ethical clearance was obtained from the institutional review board (IRB) of Jimma University, Institute of Health Science with the reference number THRPGD/502/2019. An official letter was written from the school of Nursing and Midwifery. Other necessary permissions were obtained from Jimma Medical Center (JMC). Written and verbal consent was obtained from each participant after a thorough explanation of the purpose and procedures of the study. Participation in the study was voluntary. All responses were kept confidential and anonymous.

## Results

**Socio-demographic characteristics of the study participants**: A total of 405 mothers participated; giving a response rate of 99.7%. The majority of the respondents or out of the 405 mothers who were interviewed, 234(57.8%) of the mothers resided in urban areas. 129(31.9%) of them were in the age range of 25 to 29, and the mean age (±SD) was 25.58 (±5.34) (Table 1).

**Table 1:** Socio-demographic characteristics of respondents at JUMC, 2019 (n=405)

Variables	Categories	Frequencies N=405	Percentage
Residency	Urban	234	57.8
	Rural	171	42.2
Age of mothers	15–19	50	12.3
	20–24	116	28.6
	25–29	129	31.9
	30–34	75	18.5
	35–39	31	7.7
	>=s40	4	1
ethnicity	Oromo	321	79.3
	Amhara	38	9.4
	Keffa	10	2.5
	Tigre	10	2.5
	Others^1^	26	6.3
Religion	Muslim	258	63.7
	Orthodox	100	24.7
	Protestant	47	11.6
Husband's education	Can't read and write	112	27.7
	Primary Education	124	30.6
	Secondary Education	52	12.8
	Tertiary Education	117	28.9
Mother's occupation	Unemployed^3^	350	86.4
	Employed	55	13.6
Monthly income(ETB)	Low	199	49.2
	Medium	26	6.4
	High	180	44.4
Head of house hold	Husband	349	86.2
	Self	27	6.7
	Father	3	0.7
	Others^2^	26	6.4
Decision maker	Husband	287	70.9
	Self	68	16.8
	Family	50	12.3

**Note:** others^1^-, Dawro, Yem others^2^-relatives, living in the mission, 3 unemployed-house wife

**Obstetric characteristics of respondents**: From the 405 mothers who were interviewed, 264(65.3) were multipara. Of all study participants, 355 (87.4%) had ANC follow-up and the majority, 260(56.8%) of mothers made ANC follow-up at the health center (Table 2).

**Table 2:** obstetric characteristics of respondents at JUMC, April 2019

Variables	Category	Frequency(N=405)	Percent
Parity	Primipara	141	34.7
	Multipara	264	65.3
History of Abortion	No	351	86.7
	Yes	54	13.3
ANC follow up	Yes	355	87.4
	No	50	12.6
Place of ANC follow up n=355	Hospital	111	31.2
	Health center	230	64.8
	Health post	6	1.7
	Private clinic	8	2.3
Number of ANC visit n=355	>=4 visit	220	62.0
	<4 visit	135	38.0
Planned pregnancy	Yes	288	71.1
	No	117	28.9
Maternal knowledge on danger sign and birth preparedness	Good	70	17.3
	Poor	335	82.7
Labour start time	Night	253	62.5
	Day	152	37.5

**Delay in reaching for institutional delivery service utilization**: Among a total of 405 participants, 163(40.2%) of them were delayed in reaching care for institutional delivery. As to means of transportation, 236(58.3%) mothers were traveled by ambulance; 126(31.10%) by Taxi/bus rent; 19(4.7%) by private car, 14(3.5%) on foot, 7(1.7%) of mothers were Carried by a stretcher, and the remaining 3(0.7%) were traveled by cart (Table 3).

**Table 3:** Transportation means of respondents at JMC, April, 2019

Means of transportation	frequency	Percent
Ambulance	236	58.3
taxi/bus rent	126	31.1
private car	19	4.7
On foot	14	3.5
carried by stretcher	7	1.7
Other^1^	3	0.7

Other^1^- cart

**Factors associated with delay in reaching institutional delivery service utilization among mothers at JMC**: Binary logistic regression was done accordingly and those variables with a p-value of ≤0.25 were taken to multivariable logistic regression. Residence, educational status of the mother, educational status of the husband, occupation of mother, occupation of husband, monthly income, decision-maker, parity, ANC, labor start time, status of pregnancy plan, distance, knowledge of mothers on danger sign and birth preparedness, means of transportation were variables candidates for multivariable logistic regression analysis. Multivariable logistic regression was done after identifying those candidate variables with a P-value ≤of 0.25. The goodness of fit was checked by Hosmer and Lemeshow model fitness.

The odds of mothers having a delay in reaching utilization of institutional delivery among whose husband's cannot read and write and with primary education was about 4 times higher than those mothers whose husbands educational status is in tertiary level (AOR=4.22,95% CI:1.10–16.19), (AOR=3.88,95%CI:1.24–12.1). Mothers who lived in rural areas were 2 times more likely to delay in reaching institutional delivery than those mothers who lived in urban areas (AOR=2.22,95%CI: 1.044–4.73). Mothers who live far from the health facility (distance >10 KM) were about 13 times delayed in reaching than those mothers who live ≤ 10KM from the health facility (AOR= 12.89,95%CI:6.66–24.94): (Table 4).

**Table 4:** Factors associated with delay in reaching institutional delivery service utilization among mothers at JMC, April 2019

Variables	Delay in reaching for delivery service		COR(95%CI)	AOR(95%CI)	P-value
				
	No Delay in reaching care (%)	Delay in reaching care (%)			
Husband educational status					
Can't read and write	32(28.6)	80(71.4)	24.09[11.45–50.69]	4.22[1.10–16.19]	.036*
Primary	63(50.8)	61(49.2)	9.33[4.57–19.05]	3.88[1.24–12.12]	.020*
Secondary	41(78.8)	11(21.2)	2.59[1.04–6.42]	0.61[0.15–2.50]	.491
Tertiary	106(90.6)	11(9.4%)	1	1	
Residence					
Urban	182(77.8)	52(22.2)	1	1	
Rural	60(35.1)	111(64.9)	6.48[4.17–10.05]	2.22[1.04–4.73]	.038*
Distance from health facility					
<=10km	208(84.2)	39(15.8)	1	1	
>10km	31(20.5)	120(79.5)	20.65[12.25–34.81]	12.89[6.66–24.94]	<.001*

**Note**: *P-value<0.05 AOR: adjusted odds ratio, CI: confidence interval, COR: crude odds ratio

## Discussion

Maternal delay in reaching obstetric care is one of the most contributing factors for maternal mortality and morbidity (16). The findings of this study revealed that 40.2% of mothers had a delay in utilization of service. This figure is higher in Bahirdar, Northern Ethiopia, which was 31.7% (11). On the other hand, this finding was lower in Afghanistan stating that 80% of participants had delays (12). The reason might be sample size and geographical variation. Although there were problems with transportation, the majority of this study participants used an ambulance as a means of transportation which aids mothers to reach early to a health facility. A study conducted on barriers to utilization of services in Uganda shows that factors including insecurity, poverty, and long distance from health facilities remained predictors (13).

Husband's educational status was associated with maternal delay in reaching: as husbands educational level increases, the odds of mothers to have delay decreases; this finding was consistent in Hadiya, Southern Ethiopia (9). This could be explained by as husbands' educational status increases, they will have more awareness about obstetric care.

Place of residency was the other factor associated with delay in reaching. Living in a rural area was highly associated with a maternal delay in reaching institutional delivery. According to the 2011 Ethiopian Demographic and Health Survey study participants, the major barriers for pregnant women to access health services were lack of transportation and distance to a health facility (14). In this contrast, study done in Arsi, southeast Ethiopia, we did not find that ANC is a significant contributor to maternal delay in reaching institutional delivery service utilization (21).

The likelihood of having a delay in reaching was higher among mothers who live with a distance of greater than ten kilometers far away from the health facilities than mothers who live with a distance less than or equal to ten kilometers from the health facility. Not only in Ethiopia distance is a significant barrier to institutional birth in India, even mothers who live farther from health facilities are less likely to give birth at a health facility (15). It is the major factor in delaying mothers to reach institutional delivery service utilization: women who traveled the shortest distance had a high chance of utilize delivery, whereas women who were traveling long distances had little chance to get treatment.

To sum up this study, the prevalence of delay in reaching for institutional delivery service utilization was high in JMC. The husband's educational status, residency, and distance from the health facility were the major predictors. Thus, efforts should be made to establish a strong health facility with delivery service in a rural area and need to avail within less distance of the home of mothers. The need to promote adult education as a husband's educational status was one of the significant factors.
